# Comparison of myocardial mechanical properties in patients with dilated cardiomyopathy with and without acute heart failure

**DOI:** 10.1186/1532-429X-16-S1-P239

**Published:** 2014-01-16

**Authors:** Naila Choudhary, Gangadhara Kabbli, Lynette J Duncanson, Michael Passick, Kathy Halloran, Jie J Cao

**Affiliations:** 1 Cardiology, Saint Francis Hospital, Roslyn, New York, USA

## Background

Dilated cardiomyopathy (DCM) is characterized by impaired myocardial contractile function and/or diastolic relaxation in the setting of dilated left or both ventricles that ultimately leads to heart failure (HF). In this study, we aim to compare myocardial mechanical properties in DCM patients with and without acute HF.

## Methods

We prospectively enrolled 20 patients with DCM and 8 normal controls. Of the 20 patients with DCM, 10 patients had acute HF, defined as B-type natriuretic peptide (BNP) greater than 400 pg/ml, and the remaining 10 patients were determined to have no acute HF with BNP predominantly < 100 pg/ml. All subjects underwent cardiac MRI using 1.5-T scanner. Left ventricular end-diastolic pressure (LVEDP) was assessed non-invasively using mean left atrial circulation transit time normalized by RR-interval during the first pass perfusion imaging. LV circumferential strain (CST) and strain rate (CSR) of mid LV in short axis plane and longitudinal strain (LST) and strain rate (LSR) in 4-chamber view were analyzed in cine images using feature tracking (CIM software, Auckland, New Zealand).

## Results

LV ejection fraction (EF) was significantly reduced in acute HF and in no acute HF patients compared to normal controls; 22 ± 7%, 39 ± 9% and 55 ± 2% respectively (p < 0.001). Median BNP (range) was 885 (1560) pg/ml in acute HF group, 26 (266) pg/ml in no acute HF group and 15 (48) pg/ml in normal controls (p < 0.001). Estimated LVEDP was 22 ± 12 mmHg in acute HF, 15 ± 7 mmHg in no acute HF and 8 ± 2 mmHg in normal controls (p = 0.001). Using Pearson correlation, reduced CST, CSR, LST and LSR correlated highly with reduced EF; r = -0.892, -0.790, -0.918, -0.890 (all p <0.001). The magnitude of CST, CSR, LST and LSR reduction was similar to LVEF reduction: 29% in no acute HF and 59% in acute HF group using normal controls as reference (Table 1). In contrast, the reduction of relaxation rate in early diastole in no HF patients was nearly as great as in acute HF patients (Table 1).

**Table 1:** Comparison of Strain and Strain Rate In Dilated Cardiomyopathy Patients With and Without Acute Heart Failure (HF)

Mechanical Characteristics	Groups	Mean ± SD	P value	Change (%)
CST (%)	Controls No HF HF	-14.6 ± 3.4 -9.4 ± 2.7 -5.2 ± 1.8	<0.001	- 36 65

CSR (%/second)	Controls No HF HF	-86.8 ± 20.9 -66.5 ± 20.5 -31.4 ± 10.7	<0.001	- 23 64

Rate of Circumferential Early Diastolic Relaxation (%/second)	Controls No HF HF	56.3 ± 13.2 27.2 ± 9.6 21.9 ± 12.1	<0.001	- 52 61

LST (%)	Controls No HF HF	-14.1 ± 1.6 -10.3 ± 2.6 -6.1 ± 2.2	<0.001	- 27 57

LSR (%/second)	Controls No HF HF	-65.9 ± 9 -58.5 ± 11.9 -29.8 ± 11	<0.001	- 11 55

Rate of Longitudinal Early Diastolic Relaxation (%/second)	Controls No HF HF	46 ± 8 26.7 ± 13.4 19.7 ± 11.1	<0.001	- 42 57

CST: circumferential strain of mid left ventricle in short axis plane, CSR: circumferential strain rate of mid left ventricle in short axis plane, LST: longitudinal strain in 4-chamber view, LSR: longitudinal strain rate in 4-chamber view

## Conclusions

CST, CSR, LST and LSR reduction were highly dependent on LVEF reduction suggesting load dependence of the indices. Despite marked differences in strain and strain rate reduction between patients with and without acute HF, the reduction of relaxation rate in early diastole was similarly high in both groups underscoring the significance of diastolic impairment in DCM patients which is relatively independent of HF status.

## Funding

None.

